# Animal Models of C-Reactive Protein

**DOI:** 10.1155/2014/683598

**Published:** 2014-04-24

**Authors:** Michael Torzewski, Ahmed Bilal Waqar, Jianglin Fan

**Affiliations:** ^1^Department of Laboratory Medicine, Robert Bosch Hospital, Auerbach Street 110, 70376 Stuttgart, Germany; ^2^Department of Molecular Pathology, Interdisciplinary Graduate School of Medicine and Engineering, University of Yamanashi, Yamanashi 409-3898, Japan

## Abstract

As the main theme of this special issue, CRP not only is an inflammatory marker but also has diverse biological functions associated with different diseases. To investigate CRP's physiologies and their relationship with human pathological significance, it is essential to use appropriate animal models for translational research. The most popular models for the study of CRP are transgenic mice. However, researchers should be careful when extrapolating the findings derived from these animal models. This review will discuss the current concerns on CRP transgenic mice and rabbits.

## 1. CRP Mouse Models

More than 30 epidemiological studies have demonstrated a significant association between elevated serum or plasma CRP concentration and the prevalence of atherosclerotic vascular disease, the risk of recurrent cardiovascular events among those with established disease, or the incidence of first cardiovascular events among those at risk [1]. This strong base of epidemiological evidence has led to the hypothesis that CRP is both a marker of and a causal mediator for the development of atherosclerosis.

The question regarding the role of CRP in human atherogenesis is potentially clinically relevant. If CRP as a proatherogenic factor was documented, therapeutic approaches aimed at inhibiting CRP's effects in patients with atherosclerosis would obviously be of interest. Formally, experimental approaches to investigate the role of CRP in mouse models necessitated the introduction of transgenes overexpressing human or rabbit CRP to murine strains or the generation of CRP-deficient mice that have been rendered prone to atherosclerosis. The human gene, when transferred into mice, behaves as it does in man: its expression is highly inducible and tissue-specific [2]. Male CRP transgenic mice constitutively produce human CRP, with serum levels ranging between 10 and 20 *μ*g/mL levels [3] that are comparable to those considered to indicate high risk in humans [4]. Furthermore, as human CRP produced endogenously in transgenic mouse completely avoids any possible contamination or other problems associated with administration of an extraneous CRP preparation, CRP transgenic mice were considered to provide an ideal model for studying the biological activities of human CRP* in vivo*.

Consequently, during the past ten years, a plethora of mouse studies attempted to demonstrate an atherogenic effect of CRP in genetically modified mice. Unfortunately, no clear conclusion could be drawn because these studies gave controversial and contradictory results (Table 1). Rather than answering the question of whether CRP is pro- or antiatherogenic, the following key issues and problems challenging the validity of the mouse model in general were raised as discussed.

### 1.1. Is CRP an Acute-Phase Protein in Mice?

It is a widespread belief that, unlike human CRP, mouse CRP is not an acute-phase reactant, and it is synthesized in only trace amounts [3]. However, owing to methodological shortcomings, serum levels of mouse CRP might be vastly underestimated and comparable to those in humans (see below) [12]. In any case, to overcome the alleged problem of insufficient CRP synthesis in mice, a transgenic mouse that overexpresses human CRP was generated, and this model is widely used to study the role of CRP in cardiovascular disease. The very first report by Paul et al. suggested that the expression of human CRP in mice accelerates aortic atherosclerotic lesion progression, thus asserting that CRP is indeed a risk factor and an active player in atherogenesis* in vivo* [5]. However, this interpretation has been criticised vehemently [6, 8], as differences in lesion size were observed only in males and only at one time point at the end of the study. Moreover, the reported difference “was of marginal statistical significance that could be abolished by elimination of a single outlier” [8], and the use of turpentine to boost circulating CRP levels periodically, which itself might induce active inflammatory pathology in the animals studied, was poorly controlled. Accordingly, the baseline and acute-phase human CRP concentrations were extraordinarily high (100–500 *μ*g/mL), suggesting the presence of active intercurrent inflammatory pathology in the animals studied, for which no controls were reported. Taking these issues together, the difference in the turpentine-treated group cannot be ascribed specifically to human CRP because turpentine is a major nonspecific inflammatory stimulus.

### 1.2. What Is the Gender Association of Human Transgene CRP Expression? 

It was demonstrated several years ago that both constitutive and IL-6-dependent acute-phase expression of the CRP transgene in mice requires testosterone [16, 17], restricting meaningful experimental analyses of the role of CRP in cardiovascular disease to male CRP transgenic mice. Surprisingly, despite the widespread knowledge that the expression of transgenic human CRP is under strict testosterone control, female mice were repeatedly included in the respective animal studies [5, 7]. It is no wonder that female mice were not those that provided positive results if any were obtained.

### 1.3. Which Atherosclerosis-Prone Mouse Model Mimics Human-Like Hypercholesterolemia?

Knockout mice with a defect in either apolipoprotein E (ApoE^−/−^) [18] or low-density-lipoprotein receptor (LDLR^−/−^) [19] develop atherosclerotic lesions and are currently widely used as models for investigating the pathomechanisms underlying atherosclerosis. The conflicting results obtained partly suggest that the effects of CRP on atherosclerosis are dependent on the mouse model used. First of all, ApoE^−/−^ mice have far more severe hypercholesterolemia than humans, and most of their cholesterol is contained in very low-density lipoproteins (VLDL) rather than in low-density lipoproteins (LDLs) as in humans. This issue is of high relevance as the effects of CRP on lesion development may be influenced by differences in the degree and type of hyperlipoproteinemia in the mouse models and the apoE protein* per se* can alter immune responses [20]. Immune responses that may directly involve apoE include phagocytosis of apoptotic bodies, altered macrophage dynamics [21] and altered antigen presentation efficiency [22]. Thus, the effects of human CRP on mouse atherosclerotic lesion development may be masked in ApoE^−/−^ mice because they are VLDL animals and have altered immune functions.

Consequently, the ApoE^−/−^ mouse model may not be ideal for studies of human CRP in atherosclerosis. In contrast, the LDLR^−/−^ model may be superior for a number of reasons. First, atherosclerotic lesions do not develop spontaneously as in ApoE^−/−^ animals but are inducible under a Western-type diet (WTD). Second, the serum lipoprotein pattern of LDLR^−/−^ animals is characterized by high-level LDL rather than chylomicrons and VLDL (as in ApoE^−/−^ mice) and thus more closely mimics the situation in humans [6, 10, 11].

### 1.4. What Is the Functional Role of Human CRP as a Foreign Protein in Mouse? 

It is unsurprising that there are no experimental animal models of CRP function that completely replicate the human situation. Human CRP is a foreign protein in the mouse, with many uncertainties concerning its functional role in the immune system of these animals. The situation becomes even more complicated when these mice are crossed with ApoE-deficient mice that lack a fully functional complement system. The study by Reifenberg et al. uncovered the disturbing facts that the interactions among CRP, complement, and LDL, as have been delineated in humans, may not exist similarly in mice [6]. It cannot be ruled out that mouse CRP might be active, but the inability of transgenic CRP to execute one of its primary functions (complement activation) places obvious constraints on the validity of this animal model. Moreover, transgenic human CRP operating in a xenogeneic murine environment might also fail to interact with further important mouse effector molecules, such as cellular receptors, the extracellular matrix, and lipoproteins, as pointed out by a number of studies [6, 10, 12, 23]. Thus, it is difficult to determine which of the reports are valid, because the model itself encounters several problems.

To help to overcome the above-mentioned issues, Reifenberg et al. crossbred mice expressing rabbit CRP (rbCRP) onto apoE knockout animals and studied the effect on atherogenesis [6]. Expression of the rbCRP transgene is independent of gender, and an additional inflammatory stimulus is also not required. rbCRP and human CRP are similar in structure and function. Both bind phosphocholine, C-polysaccharide, polycations, chromatin, and histones, activate complement and protect mice from lethal challenges with pneumococci [24–30]. However, no marked effect on the formation of moderately advanced atherosclerotic lesions could be discerned, either in male or in female apoE knockout mice.

### 1.5. What about the CRP-Deficient Mouse?

Owing to doubts about the physiological role of genuine mouse CRP, overexpression rather than deletion of CRP was regarded as the only meaningful way to investigate the impact of CRP on murine atherogenesis. As already mentioned above, however, serum levels of mouse CRP might be vastly underestimated and comparable to those of humans, challenging the dogma of insufficient CRP synthesis in mice. Recently, a complementary approach was chosen, generating mice with targeted deletion of the CRP gene on B6.ApoE^−/−^ as well as B6.LDLR^−/−^ genetic backgrounds [12]. This approach avoids the above-mentioned xenogeneic complications of CRP overexpression. Reliable commercial reagents along with serum CRP knockout mice as a stringent negative control indicated sufficient expression of mouse CRP even in the noninduced state. On the basis of quantitative analysis of atherosclerotic lesions, the data suggested that mouse CRP may even mediate atheroprotective effects rather than having a proatherogenic role in the two most widely used mouse models of atherosclerosis. These results together with the results by Torzewski [11] add a cautionary note to the idea of targeting CRP as therapeutic intervention against progressive cardiovascular disease and point out that CRP might actually serve a physiological and primarily nonharmful function, as first proposed in 2004 [31].

### 1.6. Is There an Association of CRP with Atherothrombosis rather than Just Atheroma? 

One has to bear in mind that most of the epidemiological studies demonstrating a significant association between elevated serum or plasma CRP concentration and the prevalence of human atherosclerotic vascular disease refer not to atherosclerotic lesion development but rather to its clinical sequelae caused by plaque rupture and thrombosis. It is therefore justified to ask whether there is an association of CRP with atherothrombosis rather than just atheroma in animal models. The respective studies, however, are far from being as numerous as those on atherosclerotic lesion development. A first report showed that occlusive thrombosis was more pronounced in CRP transgenic mice than in wild-type mice [13]. More recently, it was demonstrated that there is a more exaggerated response to vascular injury in human CRP transgenic mice [14] and that this response requires complement [15]. Caution on the interpretation of these data is warranted, however, because the vascular remodeling process associated with carotid artery ligation versus atherogenesis is not the same.

### 1.7. Conclusions

In conclusion, it is evident that each one of the above-mentioned genetically engineered mouse models addresses some of the discussed key issues and problems but leaves enough unresolved problems to call the respective mouse model into question. This can be extended to any “key issue” in atherosclerosis research that mouse models have claimed to address. They have led to very little true advance and, yet more importantly, they have generated many false and confusing concepts. Thus, caution should be exercised when extrapolating observations in genetically engineered mouse models with incompletely characterized physiological alterations to the situation in human disease. Finally, it may be appropriate to say that it was worth generating these mouse model systems, but they hardly enable us to answer definitively whether or not CRP actively contributes to human atherogenesis.

## 2. Rabbits as an Alternative CRP Model for Studying Human CRP

As described above, there are many problems of using mouse models for the study of CRP's physiological functions and controversies have risen in regard to CRP's roles in atherosclerosis. To overcome these problems, alternative animals are needed; here, we focus on using rabbit models as another means of investigating CRP biology.

### 2.1. Rabbit CRP versus Mouse CRP: Which One Is Closest to Human CRP?

The molecular and physiological features of rabbit CRP more closely resemble those of human CRP compared with mouse CRP in several aspects (Table 2) [32, 33]. First, like human CRP but unlike mouse CRP, rabbit CRP acts as a major acute-phase reactant (inflammatory marker) in the plasma and thus CRP levels are increased up to ~100 mg/L upon inflammatory stimulation [34]. It is well known that, in mouse plasma, the major inflammatory marker is serum amyloid protein (SAP) rather than CRP; plasma CRP levels in mouse are normally markedly lower than those in rabbits and humans and do not fluctuate regardless of the presence of inflammation [35]. Secondly, rabbit CRP can strongly bind with plasma atherogenic lipoproteins [36], like human CRP [37]. Thirdly, CRP immunoreactive proteins are present in all types of lesion of both rabbit and human atherosclerosis [38], but no CRP was detected in the lesions of mouse. These features of rabbit CRP lead to the notion that rabbits may be an ideal model (or a better model than mouse) for examining the physiological and pathophysiological roles of human CRP [23].

### 2.2. Rabbit Atherosclerosis Models for CRP

Rabbits have been used as an excellent model for the study of human atherosclerosis because their lipoprotein metabolism and cardiovascular system are similar to those of humans [39]. Unlike mice, but like humans, rabbits have abundant plasma cholesteryl ester transfer protein, an important regulator of cholesterol transfer, and exhibit hepatic apoB100 and intestinal apoB48 synthesis, and their lipoprotein profiles are rich in low-density lipoproteins (LDL) whereas mice are deficient in cholesteryl ester transfer protein and their plasma lipoproteins are dominated by high-density lipoproteins (HDL). Rabbits are sensitive to a cholesterol-rich diet and develop atherosclerosis rapidly, whereas most strains of wild-type mice are resistant to a cholesterol-induced atherosclerosis. WHHL rabbits provide another means of studying human familial hypercholesterolemia and atherosclerosis because these rabbits are deficient in LDL receptors [40]. A decade ago, we used both cholesterol-fed rabbits and WHHL rabbits and revealed several important features of CRP and their relationship with atherosclerosis [38]. We first found that plasma CRP levels are increased in hypercholesterolemic rabbits and correlated with the severity of aortic lesion size. Secondly, we found that CRP immunoreactive proteins are present in the lesions of atherosclerosis of rabbits regardless of the lesion types. Basically, CRP is associated mainly with extracellular matrix and seldom with macrophages. CRP is also closely colocalized with apoB and the terminal complement complex, suggesting that possible interactions between CRP-apoB-complement are present in the lesions [31, 38, 41]. Thirdly, CRP is basically synthesized by the liver rather than the vascular wall (such as macrophages). The consensus is that it is hepatically synthesized CRP that regulates plasma levels of CRP [42]. Despite this, the presence of CRP deposition in the lesions of atherosclerosis sustained efforts in the cardiovascular field during the last decade to elucidate whether CRP truly constitutes another risk factor for alongside hypercholesterolemia and is indeed involved in the initiation and progression of atherosclerotic disease. If CRP is proinflammatory and atherogenic, can we target CRP for the prevention and treatment of atherosclerosis [23]? These findings obtained from rabbit studies further strengthened the notion that rabbits are an excellent model for illustrating the relationship between CRP and atherosclerosis. Nevertheless, this study using hypercholesterolemic rabbits still cannot answer the question of whether CRP is a mediator or a marker of atherosclerosis [43].

### 2.3. Human CRP Transgenic Rabbit Model

To elucidate whether high levels of plasma CRP participate in the development of atherosclerosis, our laboratory generated 2 lines of transgenic (Tg) rabbits expressing human CRP (hCRP) transgene in the liver [44]. Plasma levels of hCRP were 0.4 ± 0.13 mg/L and 57.8 ± 20.6 mg/L in these two lines of Tg rabbits, respectively. The expression of hCRP does not cause any health disorders or phenotypes (such as spontaneous atherosclerosis) in Tg rabbits. hCRP isolated from Tg rabbit plasma exhibited the ability to activate rabbit complement, suggesting that human CRP is indeed functional in Tg rabbits [44]. Using this powerful model, we compared the susceptibility of Tg rabbits to cholesterol-rich diet-induced aortic and coronary atherosclerosis with that of non-Tg rabbits. To our surprise, neither high nor low plasma concentrations of hCRP affected aortic or coronary atherosclerotic lesion formation in Tg rabbits, even though a massive amount of hCRP was detected in the lesions of atherosclerosis [44]. Therefore, high levels of plasma and lesional CRP in Tg rabbits do not enhance the development of atherosclerosis. While these results are disappointing to CRP believer, this study cannot exclude the possibility that CRP participates in other pathological processes such as thrombosis, myocardial infarction, and arthritis. On these issues, we performed a series of experiments using hCRP Tg rabbits.

Using double balloon-injury models of the femoral arteries in Tg rabbits, we demonstrated that high expression of hCRP led to enhanced thrombosis formation on the injured smooth muscle cell-rich neointima by upregulating tissue factor expression [45], suggesting that CRP mediates thrombosis. In addition to atherosclerosis, CRP along with complement activation has been shown to accelerate myocardial infarction in rats and targeting CRP can prevent CRP-induced myocardial injury [46, 47]. However, this hypothesis has not been tested in other models, including both mouse and rabbit. Using the coronary ligation method, we generated acute myocardial infarction models in Tg rabbits. In preliminary experiments, we did not find any significant roles of CRP on myocardial infarction size or plasma cardiac markers (Waqar et al. unpublished data).

### 2.4. Effects of CRP Antisense Oligonucleotides on WHHL Rabbits

The major concern about using cholesterol-fed Tg animals is that transgenic proteins (namely, human CRP) are exogenous to animals and the atherogenic lipoproteins are remnant lipoproteins, so-called *β*-VLDLs, which may complicate the evaluation of hCRP pathophysiological functions in these models [44]. To overcome this drawback, we attempted to use a therapeutic approach to inhibit endogenous CRP and then examined the CRP-lowering effect. Towards this goal, in collaboration with ISIS Pharmaceuticals, Inc., we designed and injected robust rabbit CRP antisense oligonucleotides into WHHL rabbits, which have elevated plasma CRP levels (10~20-fold higher than in wild-type rabbits) in addition to having atherosclerosis. While tremendous efforts were made in this regard, we failed to demonstrate any therapeutic effects on atherosclerosis in WHHL rabbits (see accompanied paper by Yu et al. in this special issue).

### 2.5. Conclusions

In conclusion, rabbits are a suitable model for the investigation of CRP physiology because they resemble humans in many aspects, compared with mice. Cholesterol-fed rabbits along with WHHL rabbits and hCRP transgenic rabbits offer another opportunity to elucidate CRP functions that cannot be conducted in mice. After a decade's effort using these unique models, it is time to draw a conclusion regarding the true role of CRP in atherosclerosis. Is CRP a marker or mediator of atherosclerosis? Do we still need to continue the debate? Should we treat CVD patients who have a high level of CRP? The answers are becoming clearer and clearer: without doubt, plasma CRP levels are indeed increased and CRP is intimately present in the lesions of atherosclerosis. However, the net effect exerted by CRP is not proatherogenic, while we cannot rule out the possibility that CRP is antiatherogenic or participates in other diseases. These experimental observations are also in support of the human studies reported recently [48–51]. These studies also told us that it is unlikely that CRP can be a therapeutic target for the prevention and treatment of cardiovascular diseases.

## Figures and Tables

**Table 1:** CRP in transgenic mouse models.

	Genotype	Sex	Diet	Duration	Treatment	CRP (*μ*g/mL)	Cholesterol, triglycerides (mg/dL)	Morphometry	Significance
Atherosclerotic lesion development									
Paul et al., 2004 [5]	huCRPtg^+^/ApoE^−/−^	♂, ♀	SD	15 wks, 29 wks	Turpentine	>100	400–800, 60–120	Aortic sinus, en face	*P* < 0.02 (♂): proatherogenic
Reifenberg et al., 2005 [6]	rbCRPtg^+^/ApoE^−/−^	♂, ♀	Protein-rich	20 wks, 52 wks	—	<30	400–600, 100–200	Aorta, brachiocephalic arteries	n.s.
Trion et al., 2005 [7]	huCRPtg^+^/E3L	♂, ♀	Cholesterol-rich	25 wks, 30 wks	—	<30	510–670, 100–150	Aortic sinus	n.s.
Hirschfield et al., 2005 [8]	huCRPtg^+^/ApoE^−/−^	♂	SD	12 wks, 20 wks, 56 wks	—	<30	70–820, 40–440	Aortic sinus	n.s.
Tennent et al., 2007 [9]	huCRPtg^+^/ApoE^−/−^	♂	SD	77 wks	—	1.51–15.91	101–685, 57–159	Brachiocephalic arteries	n.s.
Kovacs et al., 2007 [10]	huCRPtg^+^/LDLR^−/−^	♂	SD	15 wks, 30 wks, 40 wks, 50 wks	—	2.4–5.18	308–377, 124–199	Aortic sinus, en face	*P* < 0.05: antiatherogenic
Torzewski et al., 2008 [11]	huCRPtg^+^/LDLR^−/−^	♂	WTD	4 wks, 8 wks, 12 wks.	—	~10	1668–2555, 741–1424	Aortic sinus, en face	n.s
Teupser et al., 2011 [12]	CRP^−/−^/ApoE^−/−^, CRP^−/−^/LDLR^−/−^	♂, ♀	SD, low fat, semisynthetic diet	12 wks, 16 wks, 20 wks, 36 wks	—	~7.5	414–615, 143–327	Aortic sinus, brachiocephalic arteries, en face	n.s.

Thrombosis and neointima formation									
Danenberg et al., 2003 [13]	huCRPtg^+^/C57BL/6	♂	SD	12 wks	Femoral wire injury, photochemical injury	18–56	—	Femoral arteries, LCCA	*P* < 0.02, *P* < 0.05: prothrombotic
Wang et al., 2005 [14]	huCRPtg^+^/C57BL/6	♀	SD	28 days	Carotid artery injury	<30	—	Carotid arteries	*P* < 0.05: vascular injury (neointima formation)
Hage et al., 2010 [15]	huCRPtg^+^/C3^−/−^	♀	SD	28 days	Carotid artery injury	<30	—	Carotid arteries	*P* < 0.05: vascular injury (neointima formation)

Note: hu: human; rb: rabbit; SD: standard diet; WTD: Western-type diet; wks: weeks; nCRP: native CRP; mCRP: modified CRP; LCCA: left common carotid artery; n.s.: not significant.

**Table 2:** Comparison of CRP in different species.

	Mouse CRP	Rabbit CRP	Human CRP
M.W. (Kd)	19.5	22	21
Plasma levels	<2 mg/L	<3 mg/L >100 mg/L*	<1 mg/L >10,000 mg/L*
Activation of complement	No	Yes	Yes
Binding to plasma LDLs	**	Yes	Yes
Deposition in the lesions of atherosclerosis	No	Yes	Yes

*In acute inflammatory state.

**Wild-type mice do not have LDLs as in humans and rabbits.

Also see [6, 32–38] for details.
